# Illinois State Medical Society

**Published:** 1873-05-01

**Authors:** 


Illinois State Medical Society.—We again remind our readers that the State Medical Society meets at Bloomington, on the third Tuesday in May (the 20th).

The list of committees expected to report can be found in another part of the present number of The Examiner. We do not learn that any railroad commutation is attainable, either for the State Society or the American Medical Association at St. Louis.

